# Robot-Assisted Nerve-Sparing Excision of a Symptomatic Obturator Nerve Schwannoma: A Case Report

**DOI:** 10.7759/cureus.11468

**Published:** 2020-11-13

**Authors:** Nikolaos Liakos, Mikolaj A Mendrek, Joern H Witt, Christian Wagner

**Affiliations:** 1 Department of Urology, Pediatric Urology and Urological Oncology, St. Antonius Hospital Gronau, Gronau, DEU

**Keywords:** robot-assisted, schwannoma, obturator nerve, nerve-sparing, pelvic lymphadenectomy

## Abstract

Reports in the literature have presented the feasibility of a minimally invasive resection of retroperitoneal or pelvic schwannomas. However, there are only a few reports in the literature about a robot-assisted nerve-sparing approach towards obturator schwannomas.

We present a case of a concomitant excision of a symptomatic obturator nerve schwannoma in a patient undergoing robot-assisted radical prostatectomy with pelvic lymphadenectomy. The patient complained about an ongoing, low-grade sensory dysfunction in the left proximal thigh area, without loss of muscular function. A preoperative pelvic MRI incidentally showed a thickening of the left obturator nerve of about 1 cm. During pelvic lymphadenectomy, the thickening was identified, an axial incision was made to the nerve sheath, and a small tumor mass (9 mm x 5 mm x 3 mm) was excised, thereby decompressing the nerve fibers and simultaneously preserving the continuity of the obturator nerve. The nerve sheath was closed using a 7-0 monofilament suture. Frozen section biopsy that was undertaken during the surgical procedure excluded the presence of a malignancy.

There were no intra- or postoperative complications. Postoperatively, the patient described a temporary sensory dysfunction of the left inner-thigh area, which regressed completely. The histopathological result confirmed a benign schwannoma of the obturator nerve. In experienced hands, the robot-assisted approach appears safe and feasible as a technique to excise a schwannoma of the obturator nerve, without the need to proceed to a full nerve resection.

## Introduction

Schwannomas are benign lesions that derive from Schwann cells, which are the cells responsible for the production of the protective myelin sheath that covers peripheral nerves. These lesions include the cellular, ancient, cystic, epithelioid, melanotic, psammomatous, schwannoma with pseudoglandular elements, and plexiform varieties [1]. Like most benign tumors, they present a relatively slow growth rate and a noninvasive character. They are enclosed into a capsule and can grow up to several centimeters, compressing the surrounding anatomical structures. Less than 1% present with malignant degeneration into a peripheral nerve sheath tumor [2].

Schwannomas in the pelvic region comprise up to 3% of all schwannomas. Most of the time, they are diagnosed due to their growth and the consecutive compression of the adjacent structures. Presenting a peak between 20 and 50 years of age, and without a preference for sex or race, they are rather challenging to diagnose by means of radiological examinations (CT or MRI) as these lesions lack any specific radiological characteristics. Hence, they are often misinterpreted as malignant urinary or gynecological tumors [3-6].

In this article, we present a case of a robot-assisted resection of a symptomatic small mass within the nerve sheath of the left obturator nerve during the lymphadenectomy part of a robot-assisted radical prostatectomy.

## Case presentation

A 58-year-old patient with a histologically confirmed intermediate-risk prostate cancer [organ-confined tumor, Gleason score of 7b, bilateral involvement of the gland, prostate-specific antigen (PSA) level: 4.6 ng/ml] presented to the outpatient clinic of our department for a consultation regarding possible treatment options. His past medical history revealed two previous surgeries for a lumbar disc herniation, as well as polyarthritic changes to several joints, and a hip joint fracture. Furthermore, he complained about a low-grade dysesthesia beginning in the inguinal area and radiating to the inner surface of his left thigh. This symptom was independent of the time of the day or physical activity, and he had not been treated until the day of presentation to the clinic.

**Figure 1:**
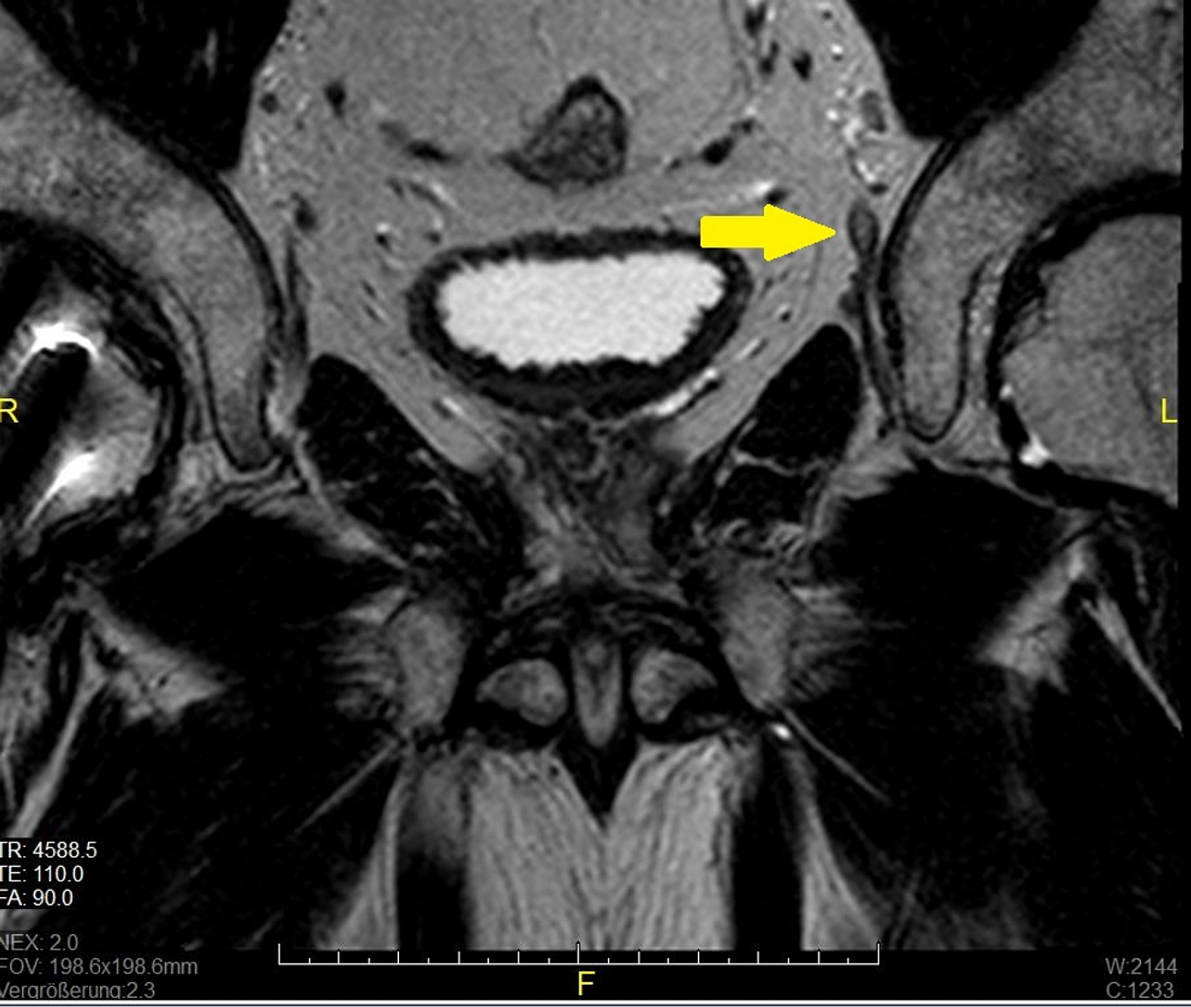
Preoperative MRI of the prostate and the pelvis The coronal sequence of the preoperative multiparametric MRI of the prostate and the pelvic area. Thickening of the neuraxon in the pars pelvica of the left obturator nerve (arrow) MRI: magnetic resonance imaging

The patient received counseling regarding prostate cancer, and he decided to undergo a robot-assisted nerve-sparing radical prostatectomy with simultaneous standard pelvic lymphadenectomy (bilateral external and internal iliac region and obturator fossa). The multiparametric MRI of the prostate and the pelvic area presented a thickening of the diameter of the neuraxon in the pars pelvica of the left obturator nerve without any specific characteristics, as shown in Figure 1. The ultrasound examination did not show any abnormal findings of the lower abdominal region. As an external compression of a nerve could not be excluded (as a cause for these symptoms), we agreed on a possible further surgical exploration in the pelvic area.

**Figure 2:**
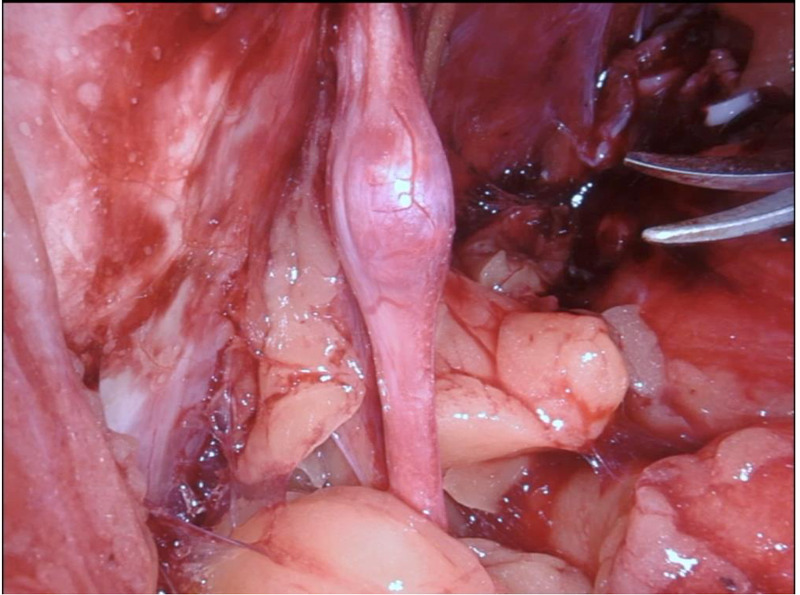
Intraoperative finding Small mass at the pars pelvica of the left obturator nerve, identified during the left-sided pelvic lymphadenectomy

**Figure 3:**
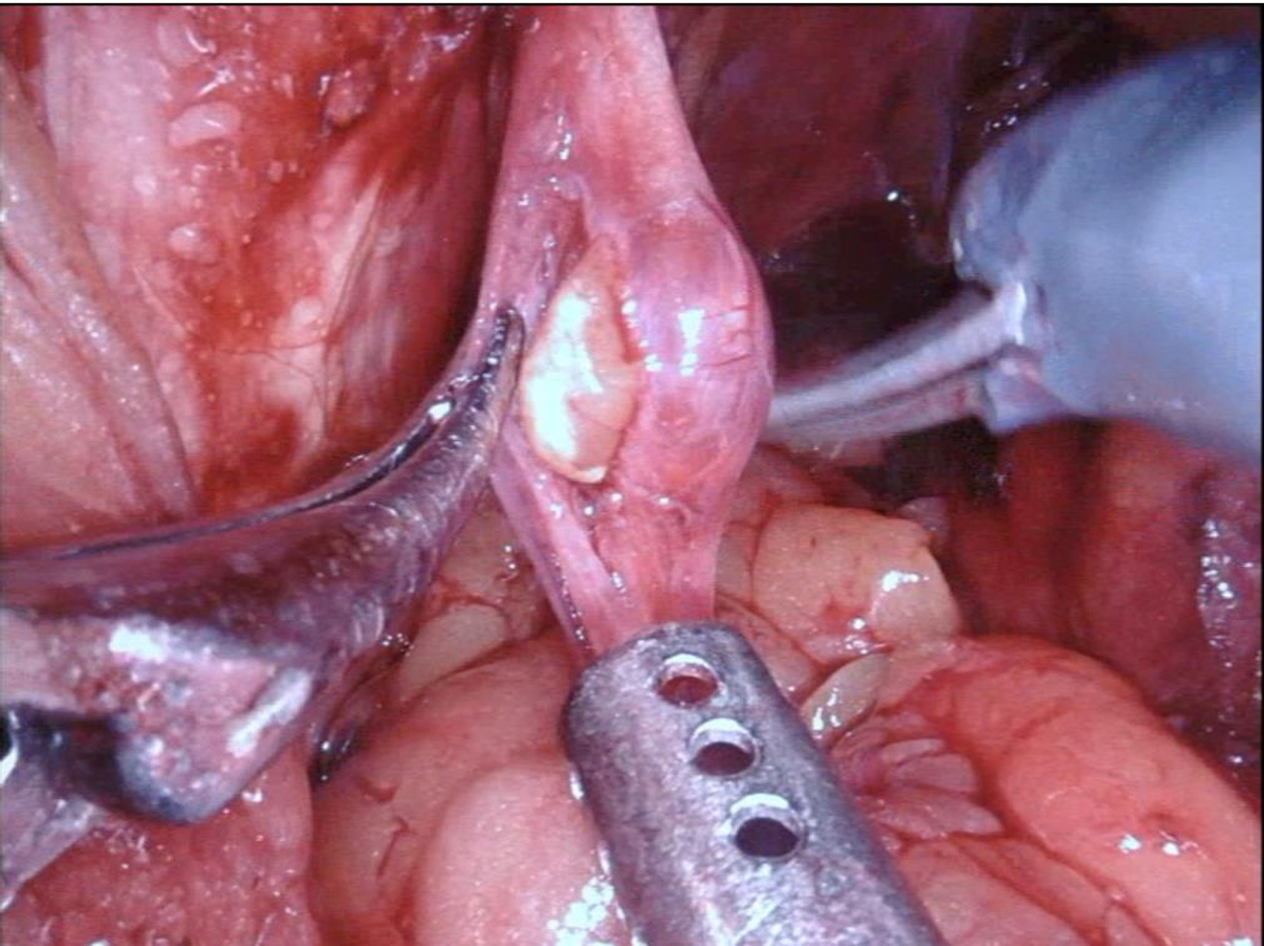
Excision of the lesion Incision of the nerve sheath with the robotic scissors with the subsequent unveiling of the schwannoma

The patient underwent an uncomplicated robot-assisted nerve-sparing radical prostatectomy. Using the standardized six-port configuration of port placement and positioning the patient in a 30° Trendelenburg position, the surgical procedure began with the transperitoneal anterior approach to the prostate gland. After the completion of the radical prostatectomy, we continued with a broad incision of the parietal peritoneum and section of the vas deferens on both sides with the aim to expose the structures in the iliac region. During the left pelvic lymphadenectomy, a small mass (9 mm x 5 mm x 3 mm) was identified at the middle intrapelvic portion of the left obturator nerve, as shown in Figure 2. A 1.5-cm longitudinal incision was made in the nerve sheath with the robotic scissors, and the yellowish mass was excised, as shown in Figure 3. The intraoperative frozen section of this lesion showed a schwannoma with benign histopathological characteristics. We proceeded to the excision of the lesion in its entirety without transection of the nerve. A neurosurgical consultation during the surgical procedure was not necessary, as there had been no need for an axial resection/graft interposition of the obturator nerve. A 7-0 resorbable monofilament suture was used for the full closure of the nerve sheath. The rest of the procedure was uneventful. The overall time for dissection, resection, and suture of the obturator schwannoma was 16 minutes; the total operative time was 210 minutes and the estimated total blood loss was 100 ml.

The final histopathological result showed peripheral nerve tissue with perineural tissue typical of a benign schwannoma, presenting cystic alterations. The entire length of hospital stay was seven days. In the immediate postoperative phase, the patient experienced a minor loss of sensitivity of the medial side of the left thigh, which resolved by the time of discharge. In the later postoperative period, there were no neurological deficits. At a follow-up nine months after the procedure, the patient did not describe any neurological deficits in the innervation area of the obturator nerve.

## Discussion

Schwannomas may grow in almost every anatomical site of the human body (peripheral nerves, visceral nerves, acoustic nerve). These benign lesions are mostly diagnosed in the cranial region as acoustic schwannomas/neurinomas and are rarely encountered in the pelvic area. Malignant degeneration is extremely uncommon (1%), and they occur in most of the cases in patients between the third and the sixth decade of life. They have been described in detail as solitary, soft lesions, and are well-differentiated from the surrounding tissue.

In contrast to literature reports about schwannomas in the retroperitoneal space, only case reports or small series of patients with schwannomas of the obturator nerve have been reported in the literature, describing mostly an open surgical or (seldom) a laparoscopical or robotic approach [7-11]. The largest cohort of patients undergoing a laparoscopical resection contained six patients, none of whom had a preoperative histological diagnosis of a schwannoma [4]. A few teams have successfully proceeded to a robot-assisted resection [10-12,13]. These publications have demonstrated the feasibility and the safety of such a tumor resection/excision with the assistance of a robotic system, sparing the continuity and the further normal function of the neural structure, as described by Perrin et al. [10].

If a tumor compresses the obturator nerve, the possible symptoms derive from the suboptimal function of the nerve (either motor or sensory deficits), which indicates a persistent external rotation of the leg with simultaneous adduction and, in many cases, a subsequent sensory loss of the inner region of the thigh. A possible partial excision of the nerve due to the adherence or invasion of the tumor can also result in these symptoms. In our case, a resection of the nerve was not necessary (without the consecutive need of an intraoperative neurosurgical consultation), as the tumor was of a rather small dimension, easy to excise, and the integrity of the nerve could be preserved. In cases of necessary resection due to the size of the mass, the involvement of a neurosurgical team for a nerve graft interposition is advisable.

## Conclusions

Pelvic schwannomas are quite rare entities and difficult to diagnose. The feasibility of a minimally invasive resection of a schwannoma in the pelvic area has been demonstrated in case reports. In this report, we demonstrated the feasibility of a minimally invasive robot-assisted technique of a nerve-sparing excision of a small obturator nerve schwannoma, utilizing frozen section biopsies during the surgical procedure.
